# 5-Cyclo­hexyl-2-methyl-3-(4-methyl­phenyl­sulfin­yl)-1-benzofuran

**DOI:** 10.1107/S1600536812008409

**Published:** 2012-03-03

**Authors:** Hong Dae Choi, Pil Ja Seo, Uk Lee

**Affiliations:** a Department of Chemistry, Dongeui University, San 24 Kaya-dong Busanjin-gu, Busan 614-714, Republic of Korea; b Department of Chemistry, Pukyong National University, 599-1 Daeyeon 3-dong, Nam-gu, Busan 608-737, Republic of Korea

## Abstract

In the title compound, C_22_H_24_O_2_S, the cyclo­hexyl ring adopts a chair conformation. The 4-methyl­phenyl ring makes a dihedral angle of 81.60 (5)° with the mean plane [r.m.s. deviation = 0.004 (1) Å] of the benzofuran fragment. In the crystal, mol­ecules are linked by weak C—H⋯O hydrogen bonds and weak π–π inter­actions between the furan rings of adjacent mol­ecules [centroid–centroid distance = 3.545 (2) Å, inter­planar distance = 3.489 (2) Å and slippage = 0.628 (2) Å.

## Related literature

For background information and the crystal structures of related compounds, see: Choi *et al.* (2011 ▶, 2012 ▶).
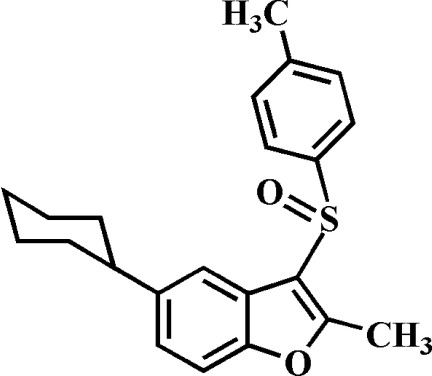


## Experimental

### 

#### Crystal data

C_22_H_24_O_2_S
*M* *_r_* = 352.47
Monoclinic, 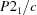

*a* = 16.6086 (4) Å
*b* = 8.8344 (2) Å
*c* = 13.0330 (3) Å
β = 104.064 (1)°
*V* = 1854.97 (7) Å^3^
*Z* = 4
Mo *K*α radiation
μ = 0.19 mm^−1^
*T* = 173 K
0.37 × 0.25 × 0.23 mm

#### Data collection

Bruker SMART APEXII CCD diffractometer
Absorption correction: multi-scan (*SADABS*; Bruker, 2009 ▶) *T*_min_ = 0.934, *T*_max_ = 0.958
16901 measured reflections
4265 independent reflections
3545 reflections with *I* > 2σ(*I*)
*R*_int_ = 0.028

#### Refinement

*R*[*F* ^2^ > 2σ(*F* ^2^)] = 0.044
*wR*(*F* ^2^) = 0.128
*S* = 1.06
4265 reflections
228 parameters
H-atom parameters constrained
Δρ_max_ = 0.59 e Å^−3^
Δρ_min_ = −0.31 e Å^−3^

### 

Data collection: *APEX2* (Bruker, 2009 ▶); cell refinement: *SAINT* (Bruker, 2009 ▶); data reduction: *SAINT*; program(s) used to solve structure: *SHELXS97* (Sheldrick, 2008 ▶); program(s) used to refine structure: *SHELXL97* (Sheldrick, 2008 ▶); molecular graphics: *ORTEP-3* (Farrugia, 1997 ▶) and *DIAMOND* (Brandenburg, 1998 ▶); software used to prepare material for publication: *SHELXL97*.

## Supplementary Material

Crystal structure: contains datablock(s) global, I. DOI: 10.1107/S1600536812008409/gk2461sup1.cif

Structure factors: contains datablock(s) I. DOI: 10.1107/S1600536812008409/gk2461Isup2.hkl

Supplementary material file. DOI: 10.1107/S1600536812008409/gk2461Isup3.cml

Additional supplementary materials:  crystallographic information; 3D view; checkCIF report

## Figures and Tables

**Table 1:** Hydrogen-bond geometry (Å, °)

*D*—H⋯*A*	*D*—H	H⋯*A*	*D*⋯*A*	*D*—H⋯*A*
C17—H17⋯O2^i^	0.95	2.48	3.140 (2)	127

Symmetry code: (i) 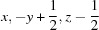.

**Fig. 1.**
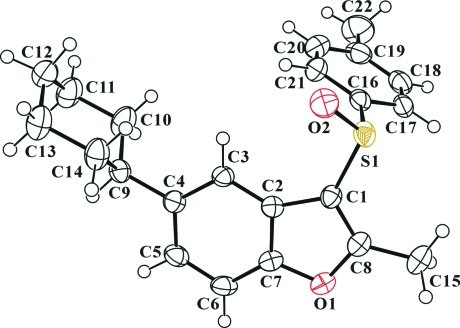
The molecular structure of the title compound with the atom numbering scheme. Displacement ellipsoids are drawn at the 50% probability level. H atoms are presented as small spheres of arbitrary radius.

**Fig. 2.**
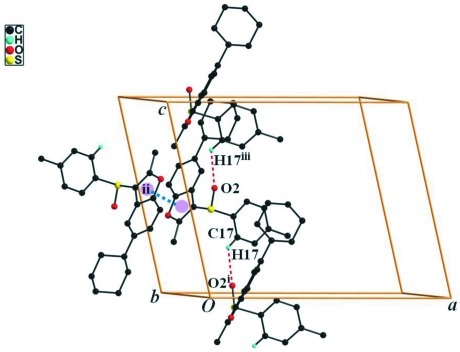
A view of the C–H···O and π–π interactions (dotted lines) in the crystal structure of the title compound. H atoms non-participating in hydrogen-bonding were omitted for clarity. [ Symmetry codes: (i) x, - y +1/2, z - 1/2; (ii) - x, - y + 1, - z + 1; (iii) x, - y + 1/2, z + 1/2.]

## supplementary crystallographic information

### Comment

As a part of our ongoing study of 5-cyclohexyl-2-methyl-1-benzofuran derivatives containing 3-(4-fluorophenylsulfinyl (Choi *et al.*, 2011) or 3-(4-bromophenylsulfinyl) (Choi *et al.*, 2012) substituents, we report herein the crystal structure of the title compound.

In the title molecule (Fig. 1), the benzofuran unit is essentially planar, with a mean deviation of 0.004 (1) Å from the least-squares plane defined by the nine constituent atoms. The cyclohexyl ring is in the chair form. The dihedral angle between the 4-methylphenyl ring and the mean plane of the benzofurn fragment is 81.60 (5)°. The crystal packing is stabilized by weak intermolecular C–H···O hydrogen bonds (Fig. 2 & Table 1). The crystal packing (Fig. 2) also exhibits weak slipped π–π interactions between the furan rings of adjacent molecules, with a Cg···Cg^ii^ distance of 3.545 (2) Å and an interplanar distance of3.489 (2) Å resulting in a slippage of 0.628 (2) Å (Cg is the centroid of the C1/C2/C7/O1/C8 furan ring).

### Experimental

77% 3-Chloroperoxybenzoic acid (224 mg, 1.0 mmol) was added in small portions to a stirred solution of 5-cyclohexyl-2-methyl-3-(4-methylphenylsulfanyl)-1-benzofuran (302 mg, 0.9 mmol) in dichloromethane (30 mL) at 273 K. After being stirred at room temperature for 4h, the mixture was washed with saturated sodium bicarbonate solution and the organic layer was separated, dried over magnesium sulfate, filtered and concentrated at reduced pressure. The residue was purified by column chromatography (hexane-ethyl acetate, 2:1 v/v) to afford the title compound as a colorless solid [yield 77%, m.p. 423-424 K; *R*_f_ = 0.52 (hexane-ethyl acetate, 2:1 v/v)]. Single crystals suitable for X-ray diffraction were prepared by slow evaporation of a solution of the title compound in ethyl acetate at room temperature.

### Refinement

All H atoms were positioned geometrically and refined using a riding model, with C–H = 0.95 Å for aryl, 1.0 Å for methine, 0.99 Å for methylene and 0.98 Å for methyl H atoms, respectively. *U*_iso_(H) =1.2*U*_eq_(C) for aryl, methine, and methylene, and 1.5*U*_eq_(C) for methyl H atoms.

### Crystal data

**Table:**

C_22_H_24_O_2_S	*F*(000) = 752
*M**_r_* = 352.47	*D*_x_ = 1.262 Mg m^−^^3^
Monoclinic, *P*2_1_/*c*	Mo *K*α radiation, λ = 0.71073 Å
Hall symbol: -P 2ybc	Cell parameters from 6233 reflections
*a* = 16.6086 (4) Å	θ = 2.5–27.5°
*b* = 8.8344 (2) Å	µ = 0.19 mm^−^^1^
*c* = 13.0330 (3) Å	*T* = 173 K
β = 104.064 (1)°	Block, colourless
*V* = 1854.97 (7) Å^3^	0.37 × 0.25 × 0.23 mm
*Z* = 4	

### Data collection

**Table:**

Bruker SMART APEXII CCD diffractometer	4265 independent reflections
Radiation source: rotating anode	3545 reflections with *I* > 2σ(*I*)
Graphite multilayer monochromator	*R*_int_ = 0.028
Detector resolution: 10.0 pixels mm^-1^	θ_max_ = 27.5°, θ_min_ = 1.3°
φ and ω scans	*h* = −21→20
Absorption correction: multi-scan (*SADABS*; Bruker, 2009)	*k* = −9→11
*T*_min_ = 0.934, *T*_max_ = 0.958	*l* = −16→16
16901 measured reflections	

### Refinement

**Table:**

Refinement on *F*^2^	Primary atom site location: structure-invariant direct methods
Least-squares matrix: full	Secondary atom site location: difference Fourier map
*R*[*F*^2^ > 2σ(*F*^2^)] = 0.044	Hydrogen site location: difference Fourier map
*wR*(*F*^2^) = 0.128	H-atom parameters constrained
*S* = 1.06	*w* = 1/[σ^2^(*F*_o_^2^) + (0.0667*P*)^2^ + 0.6552*P*] where *P* = (*F*_o_^2^ + 2*F*_c_^2^)/3
4265 reflections	(Δ/σ)_max_ < 0.001
228 parameters	Δρ_max_ = 0.59 e Å^−^^3^
0 restraints	Δρ_min_ = −0.31 e Å^−^^3^

### Special details

**Table:**

Geometry. All esds (except the esd in the dihedral angle between two l.s. planes) are estimated using the full covariance matrix. The cell esds are taken into account individually in the estimation of esds in distances, angles and torsion angles; correlations between esds in cell parameters are only used when they are defined by crystal symmetry. An approximate (isotropic) treatment of cell esds is used for estimating esds involving l.s. planes.
Refinement. Refinement of F^2^ against ALL reflections. The weighted R-factor wR and goodness of fit S are based on F^2^, conventional R-factors R are based on F, with F set to zero for negative F^2^. The threshold expression of F^2^ > 2sigma(F^2^) is used only for calculating R-factors(gt) etc. and is not relevant to the choice of reflections for refinement. R-factors based on F^2^ are statistically about twice as large as those based on F, and R- factors based on ALL data will be even larger.

### Fractional atomic coordinates and isotropic or equivalent isotropic displacement parameters (Å^2^)

**Table:**

	*x*	*y*	*z*	*U*_iso_*/*U*_eq_	
S1	0.13691 (3)	0.27171 (5)	0.44504 (3)	0.03485 (14)	
O1	0.03313 (7)	0.67453 (14)	0.40263 (9)	0.0342 (3)	
O2	0.15932 (9)	0.21178 (14)	0.55463 (10)	0.0447 (3)	
C1	0.10429 (9)	0.45997 (18)	0.45194 (11)	0.0287 (3)	
C2	0.13345 (9)	0.57018 (17)	0.53478 (11)	0.0272 (3)	
C3	0.19160 (9)	0.57274 (18)	0.63229 (11)	0.0280 (3)	
H3	0.2236	0.4854	0.6577	0.034*	
C4	0.20187 (9)	0.70563 (18)	0.69176 (12)	0.0298 (3)	
C5	0.15399 (10)	0.8332 (2)	0.65225 (14)	0.0360 (4)	
H5	0.1617	0.9233	0.6932	0.043*	
C6	0.09584 (10)	0.8331 (2)	0.55585 (14)	0.0369 (4)	
H6	0.0639	0.9203	0.5299	0.044*	
C7	0.08702 (9)	0.69938 (18)	0.49981 (12)	0.0302 (3)	
C8	0.04472 (9)	0.52723 (19)	0.37593 (12)	0.0315 (3)	
C9	0.26367 (10)	0.71423 (18)	0.79833 (12)	0.0323 (3)	
H9	0.2620	0.8199	0.8253	0.039*	
C10	0.35226 (10)	0.6834 (3)	0.79134 (14)	0.0493 (5)	
H10A	0.3679	0.7570	0.7423	0.059*	
H10B	0.3555	0.5806	0.7622	0.059*	
C11	0.41355 (11)	0.6952 (3)	0.89943 (15)	0.0522 (5)	
H11A	0.4699	0.6687	0.8924	0.063*	
H11B	0.4149	0.8008	0.9251	0.063*	
C12	0.38954 (12)	0.5904 (2)	0.97924 (15)	0.0497 (5)	
H12A	0.3949	0.4840	0.9580	0.060*	
H12B	0.4280	0.6060	1.0495	0.060*	
C13	0.30210 (13)	0.6187 (3)	0.98700 (14)	0.0520 (5)	
H13A	0.2985	0.7207	1.0171	0.062*	
H13B	0.2870	0.5435	1.0355	0.062*	
C14	0.24081 (11)	0.6080 (2)	0.87911 (13)	0.0437 (4)	
H14A	0.1845	0.6334	0.8867	0.052*	
H14B	0.2396	0.5026	0.8530	0.052*	
C15	−0.00704 (11)	0.4748 (2)	0.27292 (12)	0.0409 (4)	
H15A	0.0108	0.5256	0.2154	0.061*	
H15B	−0.0654	0.4989	0.2684	0.061*	
H15C	−0.0008	0.3651	0.2668	0.061*	
C16	0.23179 (11)	0.31164 (18)	0.40764 (12)	0.0326 (3)	
C17	0.22949 (12)	0.31441 (19)	0.30040 (12)	0.0373 (4)	
H17	0.1788	0.2968	0.2493	0.045*	
C18	0.30144 (13)	0.3429 (2)	0.26885 (13)	0.0431 (4)	
H18	0.2997	0.3467	0.1955	0.052*	
C19	0.37674 (12)	0.3662 (2)	0.34233 (15)	0.0454 (4)	
C20	0.37743 (12)	0.3619 (2)	0.44926 (15)	0.0463 (4)	
H20	0.4282	0.3778	0.5005	0.056*	
C21	0.30568 (11)	0.3349 (2)	0.48239 (13)	0.0398 (4)	
H21	0.3071	0.3323	0.5557	0.048*	
C22	0.45564 (15)	0.3941 (3)	0.3071 (2)	0.0705 (7)	
H22A	0.5017	0.4099	0.3693	0.106*	
H22B	0.4489	0.4843	0.2620	0.106*	
H22C	0.4675	0.3064	0.2672	0.106*	

### Atomic displacement parameters (Å^2^)

**Table:**

	*U*^11^	*U*^22^	*U*^33^	*U*^12^	*U*^13^	*U*^23^
S1	0.0461 (3)	0.0245 (2)	0.0314 (2)	−0.00455 (17)	0.00458 (16)	−0.00145 (14)
O1	0.0289 (5)	0.0360 (6)	0.0353 (6)	0.0024 (5)	0.0031 (4)	0.0061 (5)
O2	0.0633 (8)	0.0338 (7)	0.0361 (6)	0.0014 (6)	0.0104 (6)	0.0089 (5)
C1	0.0304 (7)	0.0267 (8)	0.0276 (7)	−0.0033 (6)	0.0042 (5)	0.0009 (6)
C2	0.0270 (7)	0.0236 (7)	0.0314 (7)	−0.0021 (6)	0.0080 (6)	0.0018 (6)
C3	0.0284 (7)	0.0230 (7)	0.0314 (7)	0.0005 (6)	0.0053 (6)	0.0007 (6)
C4	0.0264 (7)	0.0278 (8)	0.0347 (7)	−0.0033 (6)	0.0067 (6)	−0.0024 (6)
C5	0.0351 (8)	0.0258 (8)	0.0471 (9)	0.0009 (7)	0.0099 (7)	−0.0061 (7)
C6	0.0334 (8)	0.0275 (8)	0.0486 (9)	0.0079 (7)	0.0080 (7)	0.0026 (7)
C7	0.0255 (7)	0.0303 (8)	0.0343 (7)	0.0008 (6)	0.0064 (6)	0.0050 (6)
C8	0.0288 (7)	0.0343 (9)	0.0307 (7)	−0.0048 (6)	0.0063 (6)	0.0038 (6)
C9	0.0332 (8)	0.0261 (8)	0.0355 (8)	−0.0032 (6)	0.0047 (6)	−0.0063 (6)
C10	0.0301 (9)	0.0803 (15)	0.0372 (9)	−0.0076 (9)	0.0075 (7)	−0.0024 (9)
C11	0.0316 (9)	0.0774 (16)	0.0443 (10)	−0.0060 (9)	0.0027 (7)	−0.0053 (10)
C12	0.0531 (11)	0.0478 (12)	0.0410 (9)	0.0060 (9)	−0.0026 (8)	−0.0034 (8)
C13	0.0555 (12)	0.0668 (15)	0.0329 (9)	−0.0076 (10)	0.0092 (8)	−0.0018 (9)
C14	0.0402 (9)	0.0577 (12)	0.0342 (8)	−0.0097 (8)	0.0113 (7)	−0.0046 (8)
C15	0.0354 (8)	0.0531 (11)	0.0302 (8)	−0.0069 (8)	0.0001 (6)	0.0045 (7)
C16	0.0446 (9)	0.0230 (8)	0.0289 (7)	0.0044 (7)	0.0064 (6)	−0.0006 (6)
C17	0.0531 (10)	0.0277 (8)	0.0279 (7)	0.0052 (7)	0.0035 (7)	−0.0029 (6)
C18	0.0641 (12)	0.0358 (10)	0.0314 (8)	0.0111 (9)	0.0158 (8)	−0.0008 (7)
C19	0.0499 (10)	0.0419 (11)	0.0472 (10)	0.0136 (9)	0.0174 (8)	0.0010 (8)
C20	0.0422 (10)	0.0512 (12)	0.0418 (9)	0.0091 (9)	0.0033 (7)	0.0013 (8)
C21	0.0468 (10)	0.0422 (10)	0.0274 (7)	0.0066 (8)	0.0030 (7)	0.0011 (7)
C22	0.0610 (14)	0.088 (2)	0.0710 (15)	0.0125 (13)	0.0325 (12)	0.0018 (13)

### Geometric parameters (Å, º)

**Table:**

S1—O2	1.4834 (12)	C11—H11B	0.9900
S1—C1	1.7582 (16)	C12—C13	1.501 (3)
S1—C16	1.7940 (18)	C12—H12A	0.9900
O1—C8	1.373 (2)	C12—H12B	0.9900
O1—C7	1.3792 (19)	C13—C14	1.525 (2)
C1—C8	1.355 (2)	C13—H13A	0.9900
C1—C2	1.447 (2)	C13—H13B	0.9900
C2—C7	1.391 (2)	C14—H14A	0.9900
C2—C3	1.397 (2)	C14—H14B	0.9900
C3—C4	1.394 (2)	C15—H15A	0.9800
C3—H3	0.9500	C15—H15B	0.9800
C4—C5	1.403 (2)	C15—H15C	0.9800
C4—C9	1.515 (2)	C16—C21	1.384 (2)
C5—C6	1.386 (2)	C16—C17	1.389 (2)
C5—H5	0.9500	C17—C18	1.378 (3)
C6—C7	1.378 (2)	C17—H17	0.9500
C6—H6	0.9500	C18—C19	1.393 (3)
C8—C15	1.482 (2)	C18—H18	0.9500
C9—C10	1.521 (2)	C19—C20	1.391 (3)
C9—C14	1.526 (2)	C19—C22	1.510 (3)
C9—H9	1.0000	C20—C21	1.383 (3)
C10—C11	1.528 (2)	C20—H20	0.9500
C10—H10A	0.9900	C21—H21	0.9500
C10—H10B	0.9900	C22—H22A	0.9800
C11—C12	1.516 (3)	C22—H22B	0.9800
C11—H11A	0.9900	C22—H22C	0.9800
			
O2—S1—C1	107.26 (7)	C13—C12—H12A	109.4
O2—S1—C16	107.48 (8)	C11—C12—H12A	109.4
C1—S1—C16	97.46 (7)	C13—C12—H12B	109.4
C8—O1—C7	106.50 (12)	C11—C12—H12B	109.4
C8—C1—C2	107.63 (14)	H12A—C12—H12B	108.0
C8—C1—S1	123.82 (12)	C12—C13—C14	111.56 (15)
C2—C1—S1	128.54 (11)	C12—C13—H13A	109.3
C7—C2—C3	119.37 (14)	C14—C13—H13A	109.3
C7—C2—C1	104.43 (13)	C12—C13—H13B	109.3
C3—C2—C1	136.19 (15)	C14—C13—H13B	109.3
C4—C3—C2	118.92 (14)	H13A—C13—H13B	108.0
C4—C3—H3	120.5	C13—C14—C9	112.06 (15)
C2—C3—H3	120.5	C13—C14—H14A	109.2
C3—C4—C5	119.37 (15)	C9—C14—H14A	109.2
C3—C4—C9	121.06 (14)	C13—C14—H14B	109.2
C5—C4—C9	119.57 (14)	C9—C14—H14B	109.2
C6—C5—C4	122.67 (16)	H14A—C14—H14B	107.9
C6—C5—H5	118.7	C8—C15—H15A	109.5
C4—C5—H5	118.7	C8—C15—H15B	109.5
C7—C6—C5	116.24 (15)	H15A—C15—H15B	109.5
C7—C6—H6	121.9	C8—C15—H15C	109.5
C5—C6—H6	121.9	H15A—C15—H15C	109.5
C6—C7—O1	125.79 (14)	H15B—C15—H15C	109.5
C6—C7—C2	123.42 (15)	C21—C16—C17	120.55 (16)
O1—C7—C2	110.78 (14)	C21—C16—S1	121.67 (12)
C1—C8—O1	110.65 (13)	C17—C16—S1	117.76 (13)
C1—C8—C15	133.23 (17)	C18—C17—C16	119.32 (16)
O1—C8—C15	116.11 (14)	C18—C17—H17	120.3
C4—C9—C10	112.70 (13)	C16—C17—H17	120.3
C4—C9—C14	112.02 (13)	C17—C18—C19	121.36 (16)
C10—C9—C14	109.74 (15)	C17—C18—H18	119.3
C4—C9—H9	107.4	C19—C18—H18	119.3
C10—C9—H9	107.4	C20—C19—C18	118.16 (18)
C14—C9—H9	107.4	C20—C19—C22	120.8 (2)
C9—C10—C11	111.72 (15)	C18—C19—C22	121.02 (18)
C9—C10—H10A	109.3	C21—C20—C19	121.29 (17)
C11—C10—H10A	109.3	C21—C20—H20	119.4
C9—C10—H10B	109.3	C19—C20—H20	119.4
C11—C10—H10B	109.3	C20—C21—C16	119.31 (15)
H10A—C10—H10B	107.9	C20—C21—H21	120.3
C12—C11—C10	111.32 (17)	C16—C21—H21	120.3
C12—C11—H11A	109.4	C19—C22—H22A	109.5
C10—C11—H11A	109.4	C19—C22—H22B	109.5
C12—C11—H11B	109.4	H22A—C22—H22B	109.5
C10—C11—H11B	109.4	C19—C22—H22C	109.5
H11A—C11—H11B	108.0	H22A—C22—H22C	109.5
C13—C12—C11	111.39 (17)	H22B—C22—H22C	109.5
			
O2—S1—C1—C8	−146.67 (14)	C7—O1—C8—C15	−179.73 (13)
C16—S1—C1—C8	102.36 (14)	C3—C4—C9—C10	60.1 (2)
O2—S1—C1—C2	33.43 (16)	C5—C4—C9—C10	−120.06 (18)
C16—S1—C1—C2	−77.54 (14)	C3—C4—C9—C14	−64.24 (19)
C8—C1—C2—C7	−0.07 (16)	C5—C4—C9—C14	115.60 (17)
S1—C1—C2—C7	179.84 (12)	C4—C9—C10—C11	179.09 (17)
C8—C1—C2—C3	179.21 (16)	C14—C9—C10—C11	−55.3 (2)
S1—C1—C2—C3	−0.9 (3)	C9—C10—C11—C12	55.9 (3)
C7—C2—C3—C4	−0.4 (2)	C10—C11—C12—C13	−55.0 (2)
C1—C2—C3—C4	−179.61 (16)	C11—C12—C13—C14	54.7 (2)
C2—C3—C4—C5	−0.2 (2)	C12—C13—C14—C9	−55.4 (2)
C2—C3—C4—C9	179.61 (13)	C4—C9—C14—C13	−178.94 (15)
C3—C4—C5—C6	0.4 (2)	C10—C9—C14—C13	55.1 (2)
C9—C4—C5—C6	−179.46 (15)	O2—S1—C16—C21	−21.67 (17)
C4—C5—C6—C7	0.1 (3)	C1—S1—C16—C21	89.13 (15)
C5—C6—C7—O1	−179.92 (14)	O2—S1—C16—C17	156.54 (13)
C5—C6—C7—C2	−0.8 (2)	C1—S1—C16—C17	−92.67 (14)
C8—O1—C7—C6	179.88 (15)	C21—C16—C17—C18	−1.0 (3)
C8—O1—C7—C2	0.67 (16)	S1—C16—C17—C18	−179.22 (13)
C3—C2—C7—C6	1.0 (2)	C16—C17—C18—C19	1.2 (3)
C1—C2—C7—C6	−179.60 (15)	C17—C18—C19—C20	−0.8 (3)
C3—C2—C7—O1	−179.80 (12)	C17—C18—C19—C22	178.7 (2)
C1—C2—C7—O1	−0.37 (16)	C18—C19—C20—C21	0.2 (3)
C2—C1—C8—O1	0.49 (17)	C22—C19—C20—C21	−179.3 (2)
S1—C1—C8—O1	−179.43 (10)	C19—C20—C21—C16	0.0 (3)
C2—C1—C8—C15	179.27 (16)	C17—C16—C21—C20	0.4 (3)
S1—C1—C8—C15	−0.6 (3)	S1—C16—C21—C20	178.54 (14)
C7—O1—C8—C1	−0.72 (16)		

### Hydrogen-bond geometry (Å, º)

**Table:**

*D*—H···*A*	*D*—H	H···*A*	*D*···*A*	*D*—H···*A*
C17—H17···O2^i^	0.95	2.48	3.140 (2)	127

Symmetry code: (i) *x*, −*y*+1/2, *z*−1/2.
